# The Dementias Platform UK PET/MR harmonisation and test-retest study: assessment of PET repeatability and reproducibility across the national network

**DOI:** 10.1007/s00259-026-07885-4

**Published:** 2026-04-29

**Authors:** Pawel J. Markiewicz, Gerard Thompson, Joanna M. Wardlaw, Catriona Wimberley, Craig Ritchie, John-Paul Taylor, David Brooks, Ross Maxwell, Michael Firbank, Nigel Hoggard, Li Su, Jim Wild, Philip Hillel, Victoria Rhodes-Bradford, Laura M. Parkes, John T. O’Brien, Stephen F. Carter, Franklin I. Aigbirhio, Tim Fryer, Paul M. Matthews, Paresh Malhotra, Gabrielle Grey, Will Hallett, Dilek Ocal, John C. Dickson, Enrico De Vita, David L. Thomas, Nick C. Fox, Giorgios Krokos, Jane E. Mackewn, Paul Marsden, Alexander Hammers, Karl Herholz, Frederik Barkhof, Julian C. Matthews

**Affiliations:** 1 School of Computer Science and Digital Technologies, London South Bank University, London, UK; 2 Medical Physics and Biomedical Engineering, Hawkes Institute, University College London, London, UK; 3 Nuclear Medicine, Royal Free Hospital, London, UK; 4 Institute for Neuroscience and Cardiovascular Research, UK Dementia Research Institute, Edinburgh Imaging, University of Edinburgh, Edinburgh, UK; 5 School of Physics and Astronomy, University of Edinburgh, Edinburgh, UK; 6 Scottish Brain Sciences, Edinburgh, UK; 7 University of St Andrews, St Andrews, UK; 8 Translational and Clinical Research Institute, NIHR Newcastle Biomedical Research Centre, Newcastle University, Newcastle upon Tyne, UK; 9 School of Medicine and Population Health, Sheffield Institute of Translational Neuroscience, University of Sheffield, Sheffield, UK; 10 Sheffield Teaching Hospitals NHS Foundation Trust, Sheffield, UK; 11 NIHR Sheffield Biomedical Research Centre, Royal Hallamshire Hospital, Sheffield, UK; 12 Division of Neuroscience, School of Biological Sciences, Faculty of Biology, Medicine and Health, The University of Manchester, Manchester, UK; 13 School of Health Sciences, Faculty of Biology, Medicine and Health, The University of Manchester, Manchester, UK; 14 Geoffrey Jefferson Brain Research Centre, Manchester Academic Health Science Centre, Manchester, UK; 15 Department of Psychiatry, University of Cambridge School of Clinical Medicine, Cambridge, UK; 16 Department of Clinical Neurosciences, University of Cambridge School of Clinical Medicine, Cambridge, UK; 17 Department of Brain Sciences, Imperial College London, London, UK; 18 Research Department of Clinical, Educational and Health Psychology, University College London, London, UK; 19 Perceptive Inc., London, UK; 20 Dementia Research Centre, UCL Queen Square Institute of Neurology, University College London, London, London, UK; 21 Institute of Nuclear Medicine, University College London Hospital, London, UK; 22 Developmental Imaging and Biophysics Unit, Department of Developmental Neuroscience, University College London Great Ormond Street Institute of Child Health, London, UK; 23 Department of Translational Neuroscience and Stroke, UCL Queen Square Institute of Neurology, University College London, London, UK; 24 King’s College London & Guy’s and St Thomas’ PET Centre, Research Departments of Biomedical Computing and Early Life Imaging, King’s College London, London, UK; 25 School of Biomedical Engineering and Imaging Sciences, Faculty of Life Sciences and Medicine, King’s College London, London, UK; 26 Amsterdam UMC, location VU Medical Centre, Department of Radiology & Nuclear Medicine, London, The Netherlands

**Keywords:** Harmonisation, PET, PET/MR, Test-Retest, Amyloid, Multi-site, Reproducibility, Repeatability

## Abstract

**Purpose:**

Positron emission tomography combined with magnetic resonance imaging (PET/MR) has not yet achieved the level of adoption of PET/CT. This study aimed to harmonise PET imaging protocols across a national PET/MR network and to quantitatively assess whether PET/MR can achieve reliability comparable to PET/CT. While previous PET test-retest studies have demonstrated good repeatability, they have typically been limited to small cohorts or restricted site configurations.

**Methods:**

We conducted a multi-site harmonisation and rigorous test-retest study across the network of eight PET/MR scanners. Thirty-seven healthy older participants (65-90 years) underwent harmonised one-hour amyloid PET/MR scans using either [$^{18}$F]flutemetamol or [$^{18}$F]florbetaben on two occasions. Retest scans were performed under conditions of same-site repeatability or multi-site reproducibility. Harmonised acquisition and reconstruction protocols were applied, and amyloid burden was quantified on the Centiloid (CL) scale.

**Results:**

CL values across 74 scans showed excellent test-retest agreement (ICC = 0.968), improving to 0.987 after exclusion of one attenuation correction related outlier. Mean test-retest variability was 2.58%. No statistically significant differences were observed across repeatability versus reproducibility conditions, scanner types, or tracers. CL measurements were highly consistent with three independent blinded visual reads.

**Conclusion:**

This study demonstrates that harmonised PET/MR achieves high reliability comparable to PET/CT. Although the accuracy of attenuation maps requires checks, this study supports the use of PET/MR for quantitative amyloid imaging in research and therapeutic trials, and provides a valuable open resource of image and raw PET/MR data for further methodological development.

## Introduction

Positron emission tomography (PET) is an important imaging modality for the diagnosis and management of a wide range of neurological disorders [1–3]. The integration of PET with magnetic resonance imaging (PET/MR), has further expanded its capabilities by enabling the simultaneous acquisition of molecular PET data alongside high-resolution structural and functional MRI within a single session, reducing participant burden [4, 5].

In Alzheimer’s disease (AD) and related disorders, PET/MR allows the integration of established molecular biomarkers—such as amyloid-$\beta$ and tau pathology—with MRI-derived measures of neurodegeneration, facilitating the detection and characterisation of early disease processes in vivo [6–9]. This combined AT(N) biomarker classification, together with the shift towards biologically driven interventions, has become key to therapeutic trials, where PET biomarkers are used not only to define biological eligibility but also, through measurement of response, to guide treatment duration and clinical management [10–15]. Through comprehensive molecular and structural assessment, PET/MR uniquely provides essential biomarkers for patient stratification, safety monitoring (e.g., amyloid-related imaging abnormalities, ARIA), and outcome evaluation, supporting both clinical trials and emerging precision-medicine approaches [16, 17]. Despite these advantages, PET/MR has not yet achieved the same level of standardisation or widespread adoption as PET with computed tomography (PET/CT), owing to technical challenges such as MRI-based attenuation correction and the limited availability of scanners [18]. The quantitative accuracy of PET images is commonly evaluated using phantom measurements; however, in PET/MR this approach is limited by inaccuracies in attenuation correction for phantom materials [19]. When accurate attenuation maps are available, PET/MR has been shown to achieve levels of quantitative accuracy comparable to PET/CT [20]. In clinical settings, accuracy is often assessed indirectly through comparisons with PET/CT measurements [19, 21, 22], while quantitative precision is typically investigated using test-retest study designs, where variability in attenuation correction represents an additional source of measurement uncertainty. As a result, PET/MR has rarely been included in large clinical trials. To address this, a cooperative UK network of eight hybrid PET/MR scanners has been established [23] as part of the Medical Research Council funded Dementias Platform UK (DPUK, https://www.dementiasplatform.uk/) which is dedicated to advanced brain research and clinical trial delivery. The study therefore focused on improving precision through harmonisation of acquisition protocols and on evaluating this precision using a test-retest design in healthy elderly participants, with additional novel assessment of the contributing factors such as the scanner model and imaging site.

The present study aimed to: (1) harmonise PET and MR imaging protocols across the DPUK network to minimise measurement variability; (2) quantitatively assess whether PET/MR can achieve reliability comparable to PET/CT through a test-retest amyloid brain PET study; and (3) make the PET/MR image data and associated raw datasets available to the research community via controlled-access data-sharing procedures, in accordance with applicable data protection regulations. While several test-retest studies have been reported for brain PET [24–26] and whole body PET [27–29], these have typically involved either small cohorts or limited site configurations. In contrast, this study provides a comprehensive assessment of measurement variability across the DPUK network, incorporating two amyloid PET tracers and evaluating both within-site *repeatability* and across-site *reproducibility*. Such extensive assessment of reliability would not only strengthen the role of PET/MR in research studies but also support its qualification for use in therapeutic trials. Although the overall programme encompasses both PET and MRI, the present work focuses specifically on PET data quantification.

## Methods

This multi-site harmonisation and test-retest study was conducted across all eight DPUK sites equipped with hybrid PET/MR scanners consisting of three Siemens Biograph mMR systems [30] and five General Electric (GE) Signa PET/MR systems [31]. The participating sites equipped with the Siemens scanners were: (1) Edinburgh Imaging Facility QMRI; (2) University College Hospital, London; (3) King’s College London & Guy’s and St Thomas’ PET Centre. The sites equipped with the GE scanners were: (1) Campus for Ageing and Vitality, Newcastle; (2) Royal Hallamshire Hospital, Sheffield; (3) University of Manchester, Manchester University NHS Foundation Trust; (4) Wolfson Brain Imaging Centre, Cambridge (GE Signa); (5) Perceptive (formerly Invicro), Hammersmith Hospital, Imperial College London (cf. Fig. 6 in the Supplementary Information).

Healthy volunteers were recruited at each site and underwent two PET/MR scans using the same radiotracer, either [$^{18}$F]flutemetamol or [$^{18}$F]florbetaben, together with a standardised protocol of MRI sequences relevant to dementia research. Each site aimed to recruit six participants, who were first scanned at their recruiting site. The retest scan was then assigned by group randomisation to one of three conditions: (1) same-scanner *repeatability*, in which both scans were performed at the same site using the same scanner; (2) *intra-scanner-model reproducibility*, in which the retest scan was performed at a different site but using the same scanner model as the recruiting site; or (3) *inter-scanner reproducibility*, in which the retest scan was performed at a different site using a different scanner vendor.

### Participants

Ethical approval for the study was granted by the appropriate UK Research Ethics Committee (North West Greater Manchester East, 18/NW/0102), and all PET procedures were conducted under authorisation from the UK Administration of Radioactive Substances Advisory Committee (ARSAC). Healthy participants aged 65-90 years were recruited either through the Join Dementia Research platform (https://www.joindementiaresearch.nihr.ac.uk/) or through a letter of approach at participating sites. Written informed consent was obtained from all participants prior to enrolment. The study was performed in accordance with the ethical standards as laid down in the 1964 Declaration of Helsinki and its later amendments. Full details of the study’s inclusion and exclusion criteria are provided in the Supplementary Information (see Section A.3). In addition to imaging data, all study information was collected using standardized case report forms implemented within REDCap electronic data capture tools [32, 33]. Demographic and clinical variables recorded included age, sex, Addenbrooke’s Cognitive Examination-Revised (ACE-R), Mini-Mental State Examination (MMSE), Geriatric Depression Scale (GDS-15), years and level of education, medical history, previous imaging exposure, family history of dementia, and concomitant medication (Table 1). REDCap was also used to manage study logistics, including the randomisation of participants into the three test-retest groups.

**Table 1:** Participant characteristics. Continuous variables are reported as mean (SD) [range]

Characteristic	Value
Number of participants	37
Sex (F/M)	23 / 14
Age, years	72.16 (5.28) [65–82]
Years of education	16.81 (3.64) [10–25]
ACE-R score	95.65 (3.14) [88–100]
MMSE score	29.43 (0.72) [28–30]
Days between test and retest scans$^1$	63.19 (112.59) [2–482]

$^1$Due to COVID-19 and other scheduling issues, there are seven scans with periods between the test and the retest longer than 60 days

### Radiotracers

As single radiotracer supply could not reliably support all sites for the duration of the study, which was disrupted from Covid-19 shutdowns, two amyloid PET radiotracers were employed: [$^{18}$F]flutemetamol (Vizamyl™, GE Healthcare Limited) supplied by Edinburgh Imaging, University of Edinburgh, UK and GE Healthcare Limited, Amersham, UK; and [$^{18}$F]florbetaben (Neuraceq^®^, Life Molecular Imaging, Berlin, Germany; member of Lantheus) supplied by Alliance Medical Radiopharmacy Limited from sites at Guildford and Dinnington, UK. Due to the disruption, the study was scheduled in phases with each participant undergoing both test and the randomised retest scans (see Section 2 above) using the same tracer to ensure within-subject consistency. The use of two tracers was determined primarily by logistical considerations, with [$^{18}$F]flutemetamol used in Edinburgh, Newcastle, Cambridge and one London site (ICL), and [$^{18}$F]florbetaben used in Manchester, Sheffield, Cambridge, and three London sites (ICL, KCL and UCL). Radiotracers were dispensed and administered according to the manufacturers’ guidelines, with target doses of 185 MBq for [$^{18}$F]flutemetamol and 300 MBq for [$^{18}$F]florbetaben.

### PET/MR acquisition

For each of the two test-retest scanning sessions, as the MR protocol was approximately 60 minutes in duration, simultaneous PET/MR data were acquired over a 60-minute period. The target for data acquisition initiation was 60 minutes after tracer injection, ensuring that the reconstructed images encompassed the recommended static acquisition window of 90-110 minutes post-injection [34, 35]. To facilitate comparability across the network, harmonised PET and MR acquisition protocols were developed for both scanner types and implemented consistently across all DPUK sites. When feasible, participants underwent a low-dose CT scan on a PET/CT system for future comparison of attenuation-correction methods. This was performed in 16 participants at the King’s College London (KCL) site, which performed the highest number of scans across all participating sites (cf. Fig. 6).

On both Siemens Biograph mMR and GE Signa scanners, PET list-mode data were collected for 60 minutes and reconstructed into 12 frames of 5 minutes each, using the manufacturers’ implementations of ordered-subset expectation maximisation (OSEM) with 4 iterations and either 21 subsets (Siemens) or 28 subsets (GE). Although the GE system has time-of-flight capability, for fair comparison TOF information was not included in the reconstructions, nor was resolution modelling applied. Attenuation correction was performed using MR sequence-based attenuation coefficient maps ($\mu$-maps) generated by a vendor-specific methods: Brain HiRes for Siemens [36] and ZTE for GE scanners [21]. Static PET images representing the 90-110 minute post-injection interval were generated by averaging the four frames falling closest to this window. Although visually checked, these images were not corrected for motion and were analysed in the form exported directly from the scanners, reflecting typical clinical practice.

MRI acquisitions were grouped into three categories: (1) MR-based attenuation correction (MRAC) sequences used for PET attenuation correction; (2) standard structural sequences, including T$_1$-weighted MPRAGE, FLAIR, T$_2$-weighted, and T$_2$*-weighted; and (3) advanced research sequences, comprising multi post-labelling delay arterial spin labelling (ASL), multi-shell diffusion-weighted imaging (DWI), resting-state functional MRI (rs-fMRI), and quantitative susceptibility mapping (QSM). The complete MRI protocol was acquired simultaneously with the PET data. The MR images from category (1) were used to perform attenuation correction of the PET data, and the images from category (2) were read by an experienced radiologist to exclude participants with other neurological conditions. The T$_1$-weighted MRI images were used in the Centiloid processing. The image data from category (3) are not reported in this paper.

### Visual assessment

All PET image data were anonymised, standardised and prepared for independent visual evaluation by three trained readers (KH, GT, SC). Preprocessing for standardised visual reads was performed using AmyPET, an open-source Python software package for PET neuroimaging (https://amypad.github.io/AmyPET/) developed within AMYPAD, a European collaborative research initiative designed to enhance the understanding, diagnosis and management of Alzheimer’s disease through amyloid PET imaging. As a stand-alone tool, AmyPET provides the full set of functions required for image pre-processing and analysis, supporting both DICOM and NIfTI formats. For consistent blinding of visual rating, PET images and the corresponding T$_1$-weighted MPRAGE MRI were rigid-body aligned to one reference position (Montreal Neurological Institute, MNI) and resampled into a common space of 256 $\times$ 256 $\times$ 256 voxels with 1 mm$^3$ isotropic resolution. PET intensities were normalised to body weight and injected activity to generate standardised uptake value (SUV) images. The resulting PET-MRI image pairs were then anonymised further by assigning random alphanumeric codes and collated into a single dataset for blinded visual assessment. Although readers were masked to participant identity and test-retest status, they were informed of the radiotracer used. Visual assessments were performed according to the established protocols for [$^{18}$F]flutemetamol [37] and [$^{18}$F]florbetaben [38]. For comparison with centiloid values, scans were classified through the majority read [38], i.e., as positive when at least two of the three readers rated them as such, and negative otherwise. Rater percentage agreement was calculated as the proportion of scans with unanimous visual ratings divided by the total number of scans (n = 74 ). Agreement between visual reads and quantitative PET classification is reported as percent agreement and Cohen’s $\kappa$ [39, 40]. More detailed analysis of the visual reads will be reported in a separate manuscript.

### Quantitative analysis

For both radiotracers, standard Centiloid (CL) procedures were applied to first calculate standardised uptake value ratios (*SUVr*) for acquisition windows of 90-110 minute post-injection for both PET tracers. The *SUVr* was calculated using the target masks for the whole cerebellum as the reference region and the cortical target regions provided by the Centiloid Project in the MNI image space (https://www.gaain.org/centiloid-project), enabling harmonised quantification and cross-study comparability of amyloid burden as widely adopted in clinical trials and research [41]. The tracer-specific linear transformations were then used to convert the *SUVr* values to the Centiloid scale [42]. All CL calculations were performed using AmyPET software (for validation of the CL pipeline see Appendix Section D). At one Siemens site, list-mode data acquired using [$^{18}$F]florbetaben were not archived on four separate scans, and only a single histogrammed dataset was available for the entire 60-minute acquisition, resulting in a single image for the whole acquisition. To address this limitation, the desired image data were estimated within the CL procedure (level-2 calibration) using predictive linear regression modelling based on reference datasets acquired successfully with [$^{18}$F]florbetaben under full list-mode conditions [42]. See Supplementary Information for details (Section B.4).

### Statistical analysis

Global test-retest variability of CL values was evaluated using correlation analyses and Bland-Altman plots. The distributions of test-retest CL difference, $\Delta = (CL_{TP0} - CL_{TP1})$, as well as statistics such as the intraclass correlation coefficient, ICC(3,1) [29, 43], test-retest variability, $TRV = 2\times |SUVr_{TP0} - SUVr_{TP1}| / (SUVr_{TP0} + SUVr_{TP1})$^1^, and within-subject standard deviation, $s_w$ [44], were analysed. The following group differences were investigated: (i) the three categories of test-retest, including *repeatability* using the same scanner and imaging site, *intra-scanner-model reproducibility* with the same scanners but varying sites, or *inter-scanner reproducibility* with different scanner sites; (ii) three scanner groups, including the GE Signa, Siemens Biograph mMR and the mixed group of the two scanners; and (iii) the two PET tracers groups of [$^{18}$F]flutemetamol and [$^{18}$F]florbetaben. The analysis involved comparing the groups using boxplots and conducting Welch’s and Levene’s tests to determine whether the means and variances in any of these groups are statistically significant from each other. In addition, a linear mixed-effects model followed by model III ANOVA was conducted without the outlier participant C1 (see Section 3.1) to assess if there are any systematic differences between CL values with respect to the test-retest visit ($\mu _i$) (which can also be modelled as a continuous covariate—see Appendix Section C), amyloid positivity ($\alpha _r$, as determined by visual rating), PET tracer ($\beta _t$), scanner ($\theta _m$), and imaging site ($\gamma _s$). The mixed effect model for predicting the outcome of amyloid load measurement $CL_{prtsmi}$ is as follows:

1 $$\begin{aligned} CL_{prtsmi} = \alpha _r + \beta _t + \gamma _s + \theta _m + \mu _i + \rho _p + \varepsilon _{prtsmi}, \end{aligned}$$

where $\alpha _r, \beta _t, \gamma _s, \theta _m, \text {and } \mu _i$ are the respective categorical fixed parameters with the corresponding indices: $p\in \{1,36\}$ representing the 36 participants, $r\in \{1,2\}$ for the clinical read (positive/negative), $t\in \{1,2\}$ representing the two tracers, $s\in \{1,8\}$ representing the eight sites, $m\in \{1,2\}$ representing the two scanners, $i\in \{1,2\}$ representing the two time points, while $\rho _p \sim \mathcal {N}(0,\,\sigma _\text {part}^{2})$ is the random intercept for each participant and $\varepsilon _{prtsmi} \sim \mathcal {N}(0,\,\sigma ^{2})$ is the residual error.

## Results

A total of 52 participants were recruited into the study, of whom 37 successfully completed both test and retest PET/MR scans (see Table 2 for a breakdown of completed scans). Twelve planned participants did not undergo any PET/MR scanning, the majority ($n = 8$) withdrawing due to reluctance to attend in-person visits most likely due to Covid-19. An additional three participants completed only a single scan: one withdrew consent, one withdrew due to ill health, and one was unable to schedule a second scan at another site.

**Table 2:** Participant split with complete test–retest scans into repeatability and reproducibility (intra-scanner-model and inter-scanner) categories. The total number of participants scanned twice was 37

Category	Scanner	Flutemetamol	Florbetaben	Total
Repeatability	GE	4	4	8
	Siemens	2	5	7
Intra-scanner	GE	2	4	6
	Siemens	0	4	4
Inter-scanner	–	4	8	12
**Total** / Planned	–	**12** / 12	**25** / 33	**37** / 45

The mean (median; range) injected activities were 174.7 MBq (180.6; 134.1 - 196.5) for [$^{18}$F]flutemetamol and 279.3 MBq (273.1; 144.1 - 326.9) for [$^{18}$F]florbetaben. The 60-minute PET/MR scanning protocol was well tolerated by all participants, and the acquired datasets were complete and appeared high quality on visual inspection, demonstrating good contrast and anatomical detail. One participant exhibited substantial head motion, which required retrospective motion correction of the first test scan and repetition of one standard MRI sequence. In the same participant for the retest scan, a scanner malfunction caused premature termination of PET acquisition, yielding a partial dataset covering the interval of 43 minutes (63-107 minutes post-injection).

### Test-Retest reproducibility

**Fig. 1:**
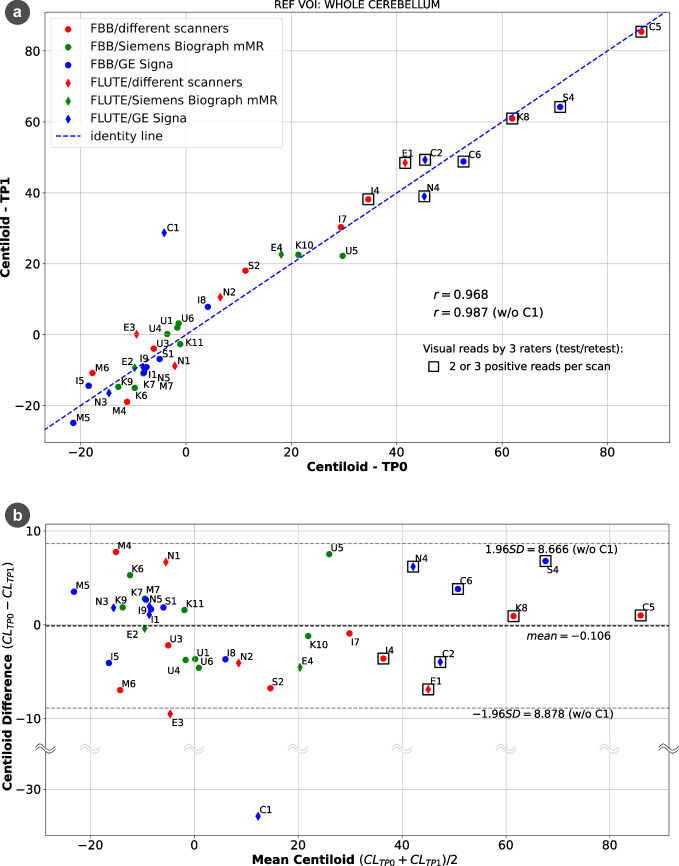
Test-retest agreement shown as scatter plot **(a)** and Bland-Altman plot **(b)**. Scanner groups are shown in different colours (red for inter-scanner, green for Siemens Biograph mMR pairs, and blue for GE Signa pairs) and the text symbols correspond to participant/site identifiers (see Table 3 in the Supplementary Information). Positive visual reads are shown in square frames based on at least two positive reads out of three per scan, obtained from three independent raters. The scans with different tracers are marked by diamonds ($\blacklozenge$) for [$^{18}$F]flutemetamol (FLUTE) and circles ($\bullet$) for [$^{18}$F]florbetaben (FBB)

Test-retest agreement for all 37 participants (74 scans in total) is illustrated using a scatter plot in Fig. 1*a* and a Bland-Altman plot in Fig. 1*b*. One participant (C1) showed notably larger test-retest differences; subsequent investigation indicated that this was attributable to PET attenuation artefact due to the inaccurate MR-derived $\mu$-map (see Supplementary Information, Section B.3). The intraclass correlation coefficient (*ICC*) for global Centiloid (*CL*) values was 0.968, improving to 0.987 after exclusion of the outlier (C1). With the exception of the outlier (C1) and one border case (E3), all data points fell within the 95% limits of agreement ($\pm 1.96 \times SD$), which corresponded to approximately $\pm 9$ CL units for this dataset (Fig. 1*b*). The mean test-retest variability (*TRV*) across participants was 2.58%. The within-subject standard deviation was $s_w$ = 3.02 (see analysis of variance below).

In Fig. 1, [$^{18}$F]flutemetamol (FLUTE) scan pairs are shown as diamonds ($\blacklozenge$) and [$^{18}$F]florbetaben (FBB) scans as filled circles ($\bullet$). Marker colours indicate scanner type: red for inter-scanner pairs (different vendors), green for Siemens Biograph mMR pairs, and blue for GE Signa pairs. Outcomes of blinded visual reads by three independent readers (six reads per test-retest pair) are indicated using empty squares representing a positive read (at least two positive reads per scan). The percent complete agreement between visual raters was 85.14%. Participant identifiers consist of a site code (e.g., ‘U’ for University College London Hospitals) and a participant number (see Supplementary Information Table 3 and the graphical illustration of performed scans across the network in Fig. 6).

### Site, equipment and tracer effects

**Fig. 2:**
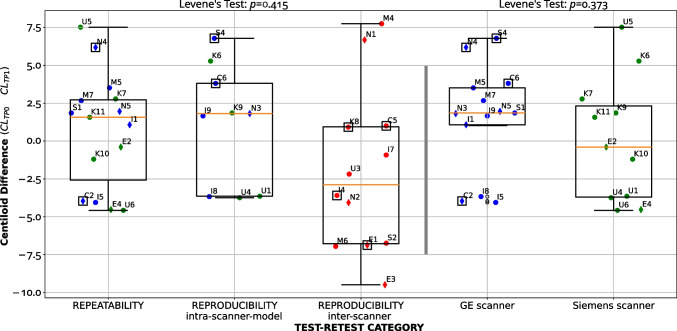
Distribution of Centiloid test-retest difference ($CL_{TP0}-CL_{TP1}$) for different test-retest categories: *repeatability* (the same scanner and site), *intra-scanner-model reproducibility* (the same scanner but different site) and *inter-scanner reproducibility* (different scanner and site), as well as intra-scanner-model comparison (within the same or different site) for GE and Siemens scanners respectively. The test-retest point for each participant is encoded (letter, symbol and colour) in the same way as in Fig. 1

Figure 2 compares test-retest CL differences across five acquisition categories (without outlier C1), the three randomised groups: (1) *repeatability* (the same site and scanner); (2) *intra-scanner-model reproducibility* (different sites, same scanner model); and (3) *inter-scanner reproducibility* (different sites and different scanner vendors/models); and in addition, test–retest differences obtained on the same scanner model are shown separately for (4) GE Signa and (5) Siemens Biograph mMR systems. Although the inter-scanner-model reproducibility distribution (third boxplot) appeared broader, the variance difference was not statistically significant (Levene’s test, $p = 0.415$). Likewise, despite visible differences between Siemens Biograph mMR and GE Signa distributions (fourth and fifth boxplots), no significant differences in variance or mean were observed (Levene’s test, $p = 0.373$; Welch’s test, $p = 0.376$). The corresponding test-retest variability (*TRV*) values based on *SUVr* were $2.193\%$ for Siemens Biograph mMR and $2.121\%$ for GE Signa.

**Fig. 3:**
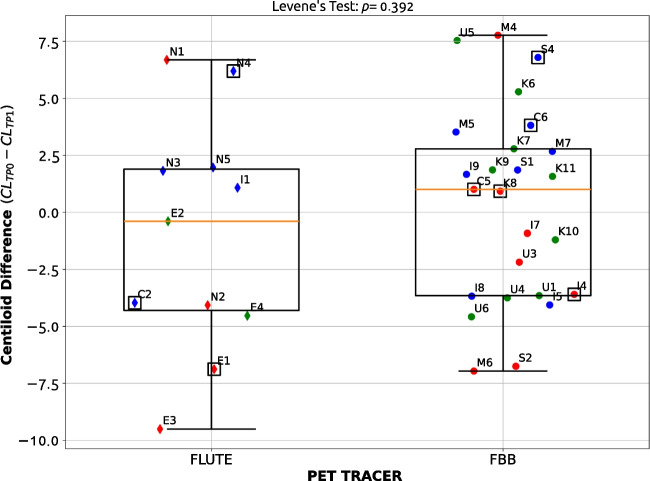
Boxplots representing the distributions of the Centiloid difference between test-retest scans for two PET tracer groups: [$^{18}$F]flutemetamol (FLUTE) and [$^{18}$F]florbetaben (FBB). The test-retest point for each participant is encoded (letter, symbol and colour) in the same way as in Fig. 1

Figure 3 presents the boxplots of CL test-retest differences for the two PET tracers. Performance was highly comparable between [$^{18}$F]flutemetamol and [$^{18}$F]florbetaben (Levene’s test $p = 0.394$; Welch’s test $p = 0.456$). Corresponding *TRV* values were $3.24\%$ for [$^{18}$F]flutemetamol and $2.29\%$ for [$^{18}$F]florbetaben, with no significant difference in mean percentage variability (Welch’s test $p = 0.221$).

**Fig. 4:**
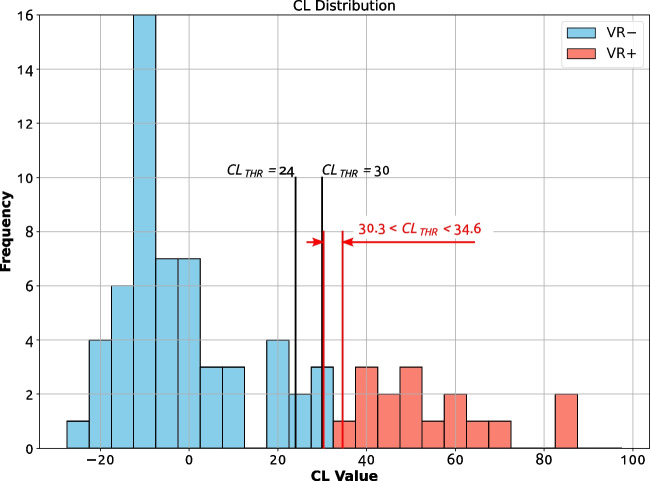
Histogram of all CL values for all 72 scans with marked negative and positive scans according to the visual reads (VR). A scan is marked negative if all three readers rated it as negative, and positive if at least two readers rated it as positive. Clear separation is achieved with a threshold of between 30.3 and 34.6 CL. The expert-consensus threshold of 24-30 CL was marked for reference

**Fig. 6:**
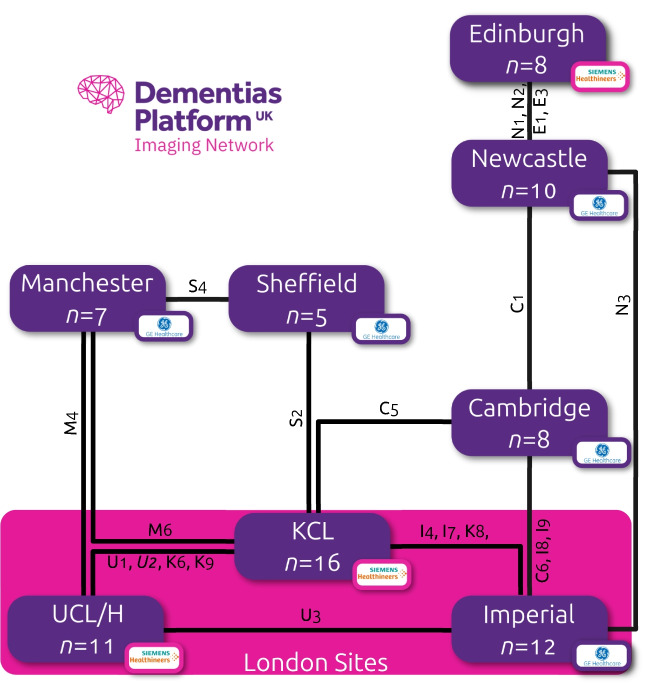
Network representation of sites and *reproducibility* test-retest scan connections. Nodes represent imaging sites and are annotated with the total number of scans performed (*n*), including 3 single scans without a corresponding retest and with badges indicating scanner vendor (Siemens Biograph mMR or GE Signa). Connection lines indicate achieved inter-site reproducibility test-retest scans, with participant codes shown adjacent to each connection (cf. Table 3)

Figure 4 illustrates the distribution of CL values for all 72 scans. Scans were classified as positive when at least two of the three readers rated them as such, and negative otherwise. This classification is consistent with the visual read markers in Figs. 1 and 2. Using this conventional classification [35, 38], a clear separation emerged between visually positive and negative scans, with a threshold of between 30.3 and 34.6 CL, achieving perfect discrimination between the two groups. The established expert-consensus threshold of 24-30 CL is marked in the figure for reference [45]. The visual-quantitative agreement for the high threshold of 30 CL was $98.61\%$ ($95\%$ CI, $92.54\%$-$99.75\%$) with the corresponding $\kappa =0.96$ ($95\%$ CI, 0.88-1.0) while for the lower threshold of 24 CL the agreement was $95.83\%$ ($95\%$ CI, $88.45\%$-$98.57\%$) with the corresponding $\kappa =0.89$ ($95\%$ CI, 0.76-1.0).

### Analysis of variance using the mixed effect model

Among all fixed effects included in the linear mixed-effects model, only visual amyloid read ($\alpha _r$) showed a statistically significant association with CL values. Specifically, visual read had a strong main effect on CL values ($F(2,41.38) = 51.22, p = 6.39\times 10^{-12}$), indicating a clear separation between amyloid-negative and amyloid-positive scans. No statistically significant effects were observed for visit ($\mu _i$), tracer ($\beta _t$), imaging site ($\gamma _s$), or scanner vendor ($\theta _m$) (all $p>0.1$), suggesting that the CL measurements were not significantly dependent on these factors.

The model showed substantial between-subject variability—the participant-level random intercept variance ($193.04; SD = 13.89$) was much larger than the residual variance ($9.10; SD = 3.02$), which corresponds to the within-subject standard deviation ($s_w$). This indicates that inter-individual differences were the dominant source of variability in CL values. Full ANOVA results and variance components are reported in Appendix Table 4. Appendix Table 5 shows alternative hierarchical modelling approach where each non-biological factor is assessed independently.

## Discussion

This Dementias Platform UK (DPUK) network study represents not only the first multi-site evaluation of harmonised PET/MR imaging protocols for dementia research but the first comprehensive repeatability and reproducibility PET study. We successfully established standardised acquisition and processing procedures across eight hybrid PET/MR sites and demonstrated excellent reproducibility of quantitative amyloid imaging metrics. These results provide a strong foundation for the use of PET/MR scanners in multi-site clinical trials and longitudinal studies of neurodegenerative disease.

The test-retest variability (*TRV*) of Centiloid (*CL*) values observed in this study was low, averaging $2.6\%$ across participants, and comparable to that reported in earlier single-centre PET/CT test-retest studies using the same two PET tracers. For example, [37] reported a *TRV* of $1.5\%$ for a composite cortical region in five participants with dementia imaged with [$^{18}$F]flutemetamol, while [24] observed a *TRV* of $4.6\%$ in a mixed group of 16 participants (eight Alzheimer’s disease and eight healthy participants) scanned with [$^{18}$F]florbetaben. Likewise, [26] reported *TRV* values between $1.85\%$ and $2.27\%$ in five participants with probable Alzheimer’s disease scanned with [$^{18}$F]flutemetamol. Using a different radiotracer, and in a multi-site setting with 20 participants, [25] demonstrated good test-retest reliability for [$^{18}$F]florbetapir, with $ICC=0.99$ in AD participants and $ICC=0.96$ in cognitively healthy participants, and *TRV* values of $2.40\%$ and $1.50\%$, respectively, across three sites, with retest scans always acquired at the same site as the initial scan. All studies, except that of [25], used a cortical cerebellar reference region to compute *SUVr*. In contrast, as in their study, we employed a whole-cerebellum reference region, which—owing to improved counting statistics and reduced segmentation variability—may offer a more stable reference for longitudinal assessments and enhance comparability across tracers and imaging sites [46, 47]. Establishing low measurement variability is essential in the era of new anti-amyloid therapies, as longitudinal amyloid PET is increasingly used to define treatment-related amyloid clearance (TRAC) to distinguish full versus partial clearance for biologically meaningful interventions [15].

Consistent with these findings, our study also demonstrated excellent test-retest reliability, with intraclass correlation coefficients of $ICC=0.97$ and 0.99 (excluding one outlier), demonstrating the robustness of CL measurements in the multi-site DPUK network. Further analyses showed that even with the more complex test-retest design, there were no statistically significant differences across repeatability, intra-scanner-model reproducibility, or inter-scanner reproducibility categories, nor across scanner types. Performance was likewise comparable between the two PET tracers, [$^{18}$F]flutemetamol and [$^{18}$F]florbetaben, highlighting the consistency of CL estimates across sites, scanners, and tracers. Among participants with longer intervals between scans (see Table 1), including those classified as amyloid-positive, there was no indication of increased test-retest differences. However, as in PET/CT, test-retest variability in PET/MR may be greater in symptomatic or more advanced disease populations.

In addition to quantitative reproducibility, we also examined the consistency of visual clinical reads across test-retest sessions. The importance of standardising visual interpretation has been implicated by the large US IDEAS study [40], which found high concordance of $86\%$ between local visual reads and centrally derived CL values across various PET tracers compared to $96\%$ in this study using the same threshold of 24 CL. As reported by [40], scans with borderline positivity ($CL=10$-40) were more likely to be discordant.

The high reproducibility demonstrated here indicates that PET/MR systems can achieve quantitative consistency comparable to PET/CT. The test-retest results are more than adequate to enable the inclusion of PET/MR scanners in multi-site clinical trials that rely on amyloid imaging for patient stratification, outcome assessment, and monitoring of therapeutic response. Importantly, these findings also highlight the feasibility of integrating PET/MR data across multiple sites and scanner models, a critical requirement for future dementia research consortia and biomarker initiatives.

Nevertheless, the accuracy of PET quantification on hybrid PET/MR systems remains dependent on robust attenuation correction as errors in MR-based attenuation maps ($\mu$-maps) can propagate to PET quantification. We observed one scan out of the 74 scans where there were significant errors (see Appendix Section B.3). These errors were clearly visible on the $\mu$-map generated using the ZTE method and resulted in much larger CL test-retest differences. For this participant, the use of an alternative atlas based attenuation correction method resulted in test-retest differences comparable to the other participants. Although not seen with the vendor-preferred Brain HiRes method on the Siemens scanner [36], some errors in $\mu$-maps were observed with alternative methods, with outliers and variability in $\mu$-map generation robustness previously reported [22]. This reinforces the need for stringent quality assurance procedures and ongoing validation of MR-based attenuation correction methods [19, 48]. Implementing systematic checks of $\mu$-maps with the availability of alternative back-up methods is therefore recommended to ensure the reliability of multi-site PET/MR studies. Nevertheless, the good test-retest results within the inter-scanner group using different implementations from two manufacturers, is suggesting good accuracy and precision in PET/MR attenuation correction.

Although time-of-flight (TOF) and resolution modelling data were acquired, these were not applied in the reconstructed images used for analysis to ensure reduced vendor-specific effects and harmonisation across sites (resolution modelling differs between vendors, and TOF capability was available only on the GE scanners). Other study limitations include the use of only two, albeit commonly employed, amyloid PET tracers and the absence of a direct head-to-head comparison with PET/CT. However, observation of Centiloid thresholds for distinguishing amyloid positive and negative scans that are comparable to those reported for PET/CT suggests that PET/MR performance in this study is broadly equivalent.

In addition to the quantitative PET metrics presented here, future work will assess the reproducibility of visual clinical reads across test-retest sessions and readers with and without the MR imaging assistance, thus providing complementary information on interpretive reliability. Further analyses will also investigate the test-retest variability of advanced MR biomarkers acquired simultaneously, such as diffusion, perfusion, and susceptibility imaging, as well as the potential influence of methodological parameters (e.g., reconstruction settings, reference regions) on reproducibility. These extensions will further strengthen the translational applicability of the harmonised DPUK PET/MR protocols in both research and clinical contexts.

## Conclusion

This study demonstrates that harmonised PET/MR achieves high reliability comparable to PET/CT. Importantly, it represents the first PET study to perform a systematic and comprehensive assessment of *inter-site*, *inter-* and *intra-scanner-model reproducibility*, extending beyond the more commonly reported test-retest repeatability studies. These findings support the use of PET/MR for quantitative amyloid imaging in research and therapeutic studies, subject to appropriate attenuation map quality checks. The openly available PET/MR images and raw data provide a resource for further methodological innovation.

## Supplementary information

This manuscript comes with Supplementary Information—see Sections A–D

## Figures and Tables

**Fig. 5:**
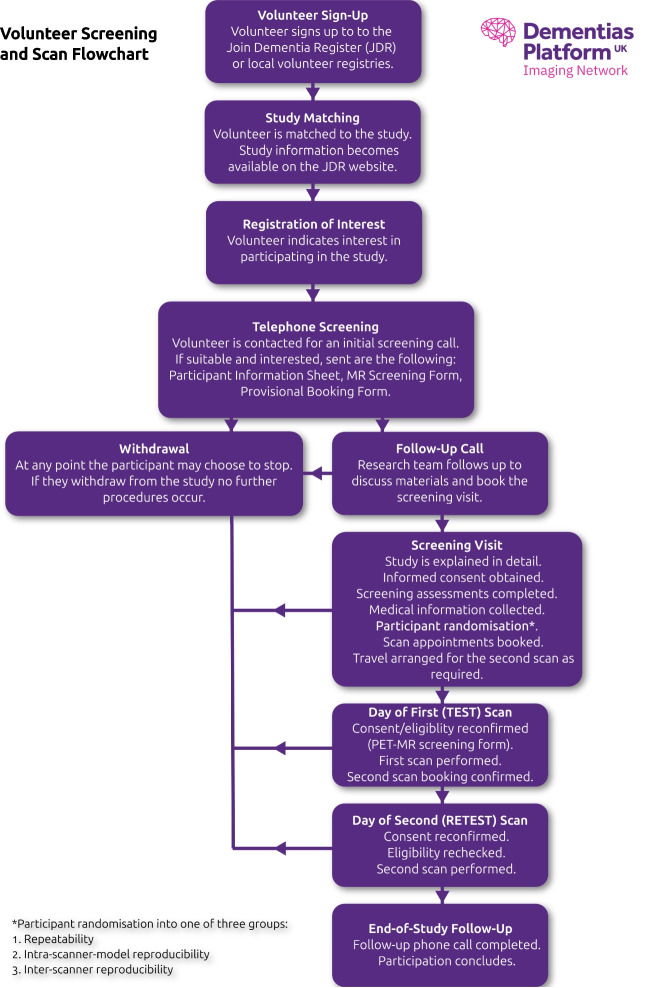
Volunteer recruitment, screening and PET/MR scan workflow

**Fig. 7:**
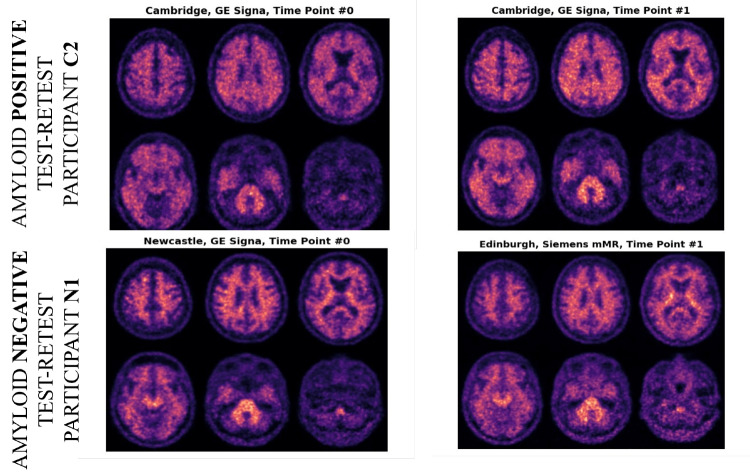
Representative reconstructed amyloid PET images acquired with [$^{18}$F]flutemetamol, shown as six transaxial slices per scan for amyloid positive (top panel) and amyloid negative (bottom panel) test-retest scans

**Fig. 8:**
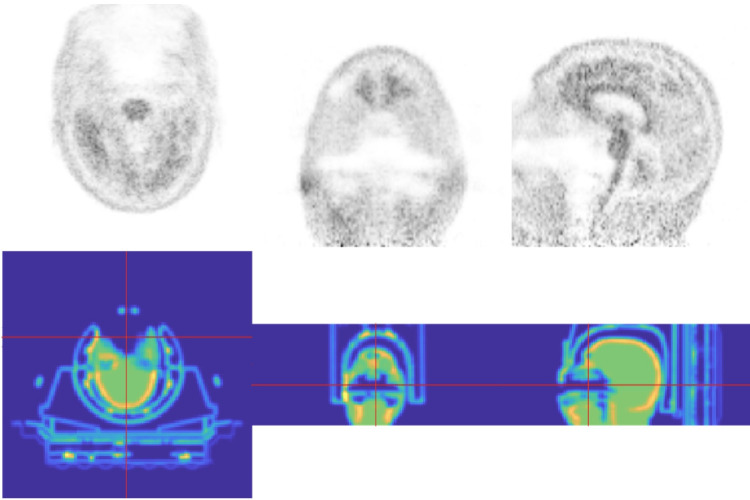
Visible artefacts in the reconstructed PET image (top, shown are transaxial, coronal and sagittal views) due to the failure of generating accurate attenuation correction map ($\mu$-map) using MR sequence (ZTE). Bottom views show the $\mu$-map images of the head, scanner bed and head coil. Unrealistically low linear attenuation coefficients were estimated particularly for the nasal region, resulting in clear artefacts around the corresponding region in the reconstructed PET image

**Fig. 9:**
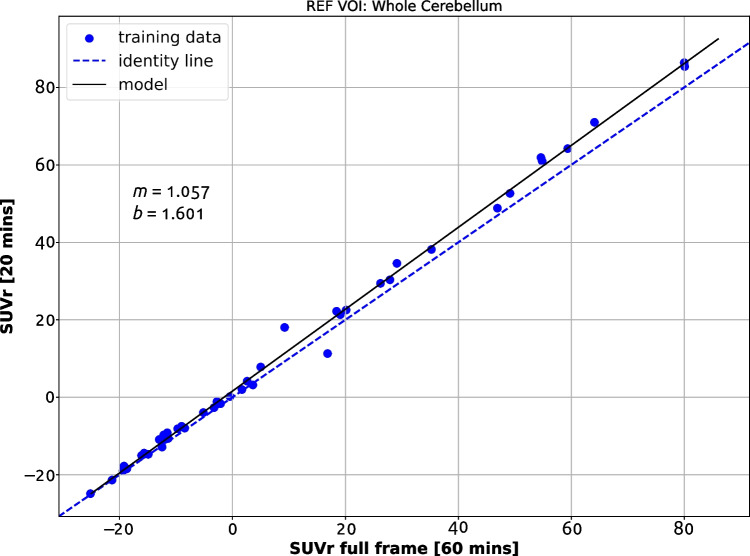
Linear regression model used to estimate missing 20-minute (90–110 min post-injection) *SUVr* values for [$^{18}$F]florbetaben scans

**Fig. 10:**
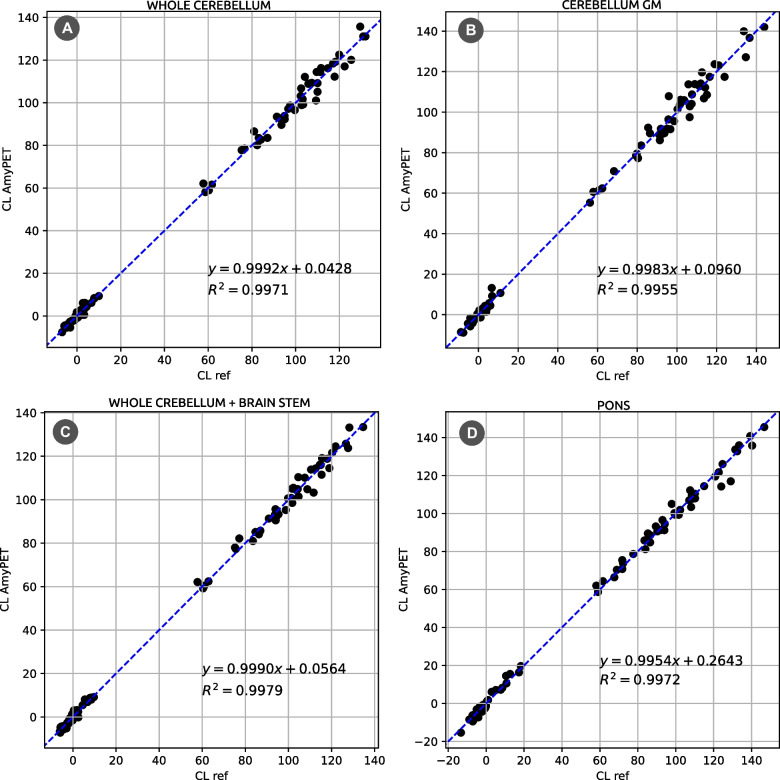
Replication of the Level-1 Centiloid standard PiB analysis using the AmyPET quantification pipeline. Scatter plots show correlations between SUVR values obtained with the present implementation and those from the Centiloid standard analysis for 34 young controls and 45 Alzheimer’s disease participants. Results are presented for four reference regions: (**A**) whole cerebellum, (**B**) cerebellar cortex, (**C**) whole cerebellum plus brainstem, and (**D**) pons. Dashed lines denote linear regression fits. Regression statistics are summarised in Table 6

## Data Availability

The image and raw PET/MR data will be available at https://www.dementiasplatform.uk/.

## Appendix A: Study information

### A.1 About the study

The study was approved by the NHS Health Research Authority (NRA) ethics committee (Ref: 18/NW/0102; IRAS 223411) and the UK Administration of Radioactive Substances Advisory Committee (ARSAC). The University of Manchester acted as the sponsor for the study.

The flowchart in Fig. 5 summarises the participant pathway from initial registration through screening, consent, and completion of two PET/MR scans. Participants could withdraw from the study at any point, including around the first scan visit.

### A.2 Participant recruitment & screening

Participants were recruited across the Dementias Platform UK (DPUK) PET/MR network through Join Dementia Research (JDR) and locally maintained volunteer registries at each participating site. Screening involved completion of cognitive assessment and questionnaires, including Geriatric Depression Scale (GDR-15), Addenbrooke’s Cognitive Examination (ACE-R), and the clinical history, taking approximately 30-40 minutes.

### A.3 Inclusion and exclusion criteria

Eligible participants were healthy older adults aged 65-90 years who were cognitively healthy at screening and able to tolerate PET/MR procedures as well as any required travel between scanning sites. Screening was used to ensure participants were able to tolerate the procedures, and to exclude neurological and psychiatric conditions that could influence imaging measurements. Although the study targeted cognitively normal volunteers, approximately 25% of participants were expected to be amyloid-positive based on previous studies in this age group. This enabled assessment of repeatability and reproducibility in both amyloid-positive and amyloid-negative cases.

Participants were excluded if they were unable to tolerate lying still in the scanner, experienced significant discomfort or claustrophobia, were unable to undergo venous cannulation, or if the study team identified safety concerns during screening. Pregnant or breastfeeding women were not eligible for participation, although all female participants were expected to be beyond child-bearing age due to the study’s minimum age requirement of 65 years. Participants were informed that, following their initial scan, they would undergo a second scan either at the same site, at another site with the same scanner model, or at a site using different scanning equipment.

### A.4 Consent and ethical approval

Consent was obtained at each of the recruiting sites by appropriately qualified and trained staff. Participants were provided with a Patient Information Sheet, given at least 24 hours to consider participation, and provided opportunities to ask questions before signing a consent form. All PET radiotracer administrations were conducted under authorisation from the UK ARSAC, with radiation exposure justified and assessed by the study’s Medical Physics Expert and Clinical Radiation Expert.

## Appendix B: Scanning information

### B.1 Scanning across the network

Scanning across the DPUK PET/MR network was conducted in three phases, driven primarily by radiotracer availability and substantially disrupted by the COVID-19 pandemic. In the initial phase, prior to COVID-19 lock-down, all participating sites were bound to use [$^{18}$F]flutemetamol for PET imaging. Post lock-down, the access to [$^{18}$F]flutemetamol was then restricted to northern sites, specifically Edinburgh and Newcastle. In the final phase, post lock-down, [$^{18}$F]florbetaben was used at the remaining sites in the south of the UK. The availability of tracers and scheduling of scans were highly affected by COVID-19–related disruptions. As shown in Fig. 6, some inter-scanner test-retest combinations were not explored due to restrictions in tracer availability at different locations, travel restrictions, and limited number of available scans. Importantly, each participant underwent both test and retest scans using the same radiotracer to ensure within-subject consistency. Table 3 shows all the network sites (listed are academic institutions, see also Fig. 6) with corresponding PET/MR scanner, the code for participants.

**Table 3:** Site name coding for participants across DPUK imaging sites. An “x” denotes a single digit representing the participant recruitment number for any site

Academic site name	Scanner at the site	Recruitment code	Participant graph code
University of Cambridge	GE Signa	CAM	Cx
The University of Edinburgh	Siemens mMR	EDI	Ex
Imperial College London	GE Signa	ICL	Ix
King’s College London	Siemens mMR	KCL	Kx
The University of Manchester	GE Signa	MAN	Mx
Newcastle University	GE Signa	NEW	Nx
The University of Sheffield	GE Signa	SHF	Sx
University College London	Siemens mMR	UCL	Ux

### B.2 Example reconstructed images

Reconstructed example amyloid PET images acquired with [$^{18}$F]flutemetamol are shown in Fig. 7 as six representative transaxial slices per scan, illustrating amyloid-positive scans (top panels) and amyloid-negative scans (bottom panels). The positive test-retest pair was acquired at the same site (Cambridge), whereas the negative test-retest pair was acquired at two different sites using different PET/MR scanner models (test scan at Newcastle and retest scan at Edinburgh), demonstrating visual comparability across multi-site acquisitions.

### B.3 Investigation of an outlier scan

An obvious artefact was observed in the retest PET image of participant CAM001, which was caused by the $\mu$-map used for attenuation correction—the attenuation correction algorithm, which uses the ZTE MR sequence, was identified as the source of the artefact (Fig. 8). Reprocessing the data using an alternative attenuation correction method resolved the issue and resulted in a good test-retest agreement.

### B.4 SUVr linear model for failed archive of raw PET data

At one Siemens PET/MR site (University College London Hospitals), list-mode data were not archived for four participants (UCL003, UCL004, UCL005, and UCL006) due to an operational failure. This issue affected baseline (test) scans only and occurred for a single tracer, [$^{18}$F]florbetaben. Although the list-mode data required for dynamic reconstruction could not be recovered, the corresponding raw sinogram data were retained, representing the full 60-minute acquisition (60-120 minutes post-injection). To allow inclusion of these participants in the test-retest analysis, the CL values for the missing 20-min (90-110 minutes) *SUVr* values were estimated using a linear regression model within a slightly modified CL procedure (level-2 calibration—see Fig. 9). This model was derived from complete [$^{18}$F]florbetaben datasets acquired at other sites and related *SUVr* values computed from the full 60-minute acquisition to those obtained from the standard 20-minute window. For the four participants with missing list-mode data, the linear fit was subsequently used to estimate the *SUVr* values within the 20 minutes window from the measured *SUVr* values within the available 60 minute window.

## Appendix C: Mixed-effects modelling

### C.1 Summary of mixed-effects modelling results

Tables 4 and 5 summarise the results of linear mixed-effects modelling of Centiloid (CL) values, evaluating the contribution of fixed and random effects to measurement variability. In the first table, a full fixed-effects model is presented, incorporating categorical fixed effects for visual amyloid read (negative/positive), visit (test/retest), PET tracer ([$^{18}$F]florbetaben vs [$^{18}$F]flutemetamol), imaging site (eight levels), and scanner model (Siemens mMR vs GE Signa), with subject included as a random effect via a random intercept to account for repeated measures. This model provides a comprehensive assessment of potential systematic sources of variation in CL values across the multi-site PET/MR network.

The second table presents a reduced set of models focused on visual read as the primary fixed effect of interest, combined with one additional fixed factor at a time (e.g., visit, tracer, site, or scanner), while retaining subject as a random intercept and a separate residual error term. This modelling strategy enables isolation of the effect of visual read as a biological factor from the non-biological factors which potentially can introduce systematic shifts in measured amyloid burden (leading to reduced harmonisation). This hierarchical modelling approach allows the contribution of each factor to be assessed independently, while preserving interpretability and avoiding overparameterisation for the limited sample size. Across models, CL value was used as the outcome variable, and all fixed effects were treated as categorical factors.

**Table 4:** Type III ANOVA results (Satterthwaite’s method) for linear mixed-effects model with all fixed effects and participant ($\rho _p$) as a random intercept. Fixed effects include visual read ($\alpha _r$), visit modelled either as a categorical factor in full model #1 ($\mu _i$) or as a continuous covariate in full model #2 (with $v_{prtsmi}$ denoting the timing variable and $\mu$ the corresponding fixed-effect parameter), PET tracer ($\beta _t$), imaging site ($\gamma _s$) and scanner vendor ($\theta _m$) (cf. Eq. 1)

Full Model #1:	$CL_{prtsmi} = \alpha _r + \beta _t + \gamma _s + \theta _m + \mu _i + \rho _p + \varepsilon _{prtsmi}$					
Effect	Sum Sq	Mean Sq	Num DF	Den DF	F value	*p*-value
$\alpha _r$	913.90	913.90	1	32.179	100.4840	$2.01\times 10^{-11}$
$\mu _i$	0.11	0.11	1	28.533	0.0122	0.9128
$\beta _t$	6.18	6.18	1	51.854	0.6790	0.4137
$\gamma _s$	112.91	16.13	7	31.637	1.7735	0.1277
$\theta _m$	14.88	14.88	1	33.836	1.6359	0.2096
Random effects	Variance					
$\rho _p$	193.044					
Residual	9.095					
Full Model #2:	$CL_{prtsmi} = \alpha _r + \beta _t + \gamma _s + \theta _m + \mu v_{prtsmi} + \rho _p + \varepsilon _{prtsmi}$					
Effect	Sum Sq	Mean Sq	Num DF	Den DF	F value	*p*-value
$\alpha _r$	896.37	896.37	1	32.184	100.4203	$2.022\times 10^{-11}$
$\mu$	5.15	5.15	1	29.955	0.5774	0.4533
$\beta _t$	5.49	5.49	1	51.642	0.6156	0.4363
$\gamma _s$	117.79	16.83	7	31.686	1.8851	0.1053
$\theta _m$	15.12	15.12	1	33.811	1.6942	0.2018
Random effects	Variance					
$\rho _p$	192.685					
Residual	8.926					
Number of observations $n=72$; Number of participants $p=36$

**Table 5:** Type III ANOVA results (Satterthwaite’s method) for various linear mixed-effects models with participant ($\rho _p$) as a random intercept (cf. Table 4 and Eq. 1)

Effect	Sum Sq	Mean Sq	Num DF	Den DF	F value	*p*-value
Model #1:	$CL_{prtsmi} = \alpha _r + \rho _p + \varepsilon _{prtsmi}$					
$\alpha _r$	986.38	986.38	1	34	98.335	$1.446\times 10^{-11}$
Model #2:	$CL_{prtsmi} = \alpha _r + \mu _i + \rho _p + \varepsilon _{prtsmi}$					
$\alpha _r$	1014.0	1014.0	1	34	98.3348	$1.446\times 10^{-11}$
$\mu _i$	0.2	0.2	1	35.000	0.0192	0.8907
Model #2b:	$CL_{prtsmi} = \alpha _r + \mu v_{prtsmi} + \rho _p + \varepsilon _{prtsmi}$					
$\alpha _r$	1010.82	1010.82	1	33.978	98.2305	$1.476\times 10^{-11}$
$\mu$	0.15	0.15	1	36.346	0.0144	0.905
Model #3:	$CL_{prtsmi} = \alpha _r + \beta _t + \rho _p + \varepsilon _{prtsmi}$					
$\alpha _r$	988.87	988.87	1	33	98.5828	$1.935\times 10^{-11}$
$\beta _t$	8.12	8.12	1	33	0.8094	0.3748
Model #4:	$CL_{prtsmi} = \alpha _r + \gamma _s + \rho _p + \varepsilon _{prtsmi}$					
$\alpha _r$	835.99	835.99	1	34.021	95.4626	$2.093\times 10^{-11}$
$\gamma _s$	108.38	15.48	7	34.770	1.7681	0.1255
Model #5:	$CL_{prtsmi} = \alpha _r + \theta _m + \rho _p + \varepsilon _{prtsmi}$					
$\alpha _r$	1027.2	1027.2	1	33	102.4090	$1.201\times 10^{-11}$
$\theta _m$	18.54	18.54	1	33	1.8483	0.1832

## Appendix D: Centiloid pipeline calibration

To ensure that application of the Centiloid transformation was appropriate, concordance between the quantification pipeline implemented in the AmyPET software and the Level-1 Centiloid standard analysis was evaluated [42]. The standard PiB dataset was processed in AmyPET following spatial normalisation to the MNI space and extraction of SUVR values using the Centiloid cortical target volume of interest (VOI) together with the four reference regions. Linear regression analyses comparing our results with those obtained using the published Centiloid standard processing results demonstrated very close agreement across all reference regions (Table 6 and Fig. 10). The observed slopes, intercepts and coefficients of determination indicate that the quantitative accuracy of AmyPET processing stream closely reproduces that of the Centiloid standard method.

**Table 6:** Level-1 agreement of AmyPET with the Centiloid standard analysis presented for the four reference regions: whole cerebellum (WC), cerebellar cortex (CG), whole cerebellum plus brainstem (WC+B), and pons

Reference VOI	Slope	Intercept	$R^2$
	(0.98 to 1.02)	(-2 to 2 CL)	($>0.98$)
WC	0.9992	0.0428	0.9971
WC+B	0.9990	0.0564	0.9979
CG	0.9983	0.0960	0.9955
Pons	0.9954	0.2643	0.9972

## Footnotes

1 The test-retest variability (*TRV*) was calculated using standardised uptake value ratios (*SUVr*) to avoid the problem of heteroscedasticity of inflating the variability estimates for near zero CL values. Since the CL values are already normalised and in the ‘centi’ scale, it is easier to report the CL difference.
